# Ongoing circulation of emerging tick-borne viruses in Poland, Eastern Europe

**DOI:** 10.1371/journal.pone.0330544

**Published:** 2025-09-03

**Authors:** Koray Ergunay, Gocha Golubiani, Giorgi Kirkitadze, Drew D. Reinbold-Wasson, Brian P. Bourke, Cody A. Phelps, Adam Kotorashvili, Nato Kotaria, Christine E. Hulseberg, Tamar Chunashvili, Andrew Sydenstricker, Anano Shubashishvili, Thomas A. Musich, Yvonne-Marie Linton

**Affiliations:** 1 Department of Entomology, Smithsonian Institution–National Museum of Natural History (NMNH), Washington, Columbia, United States of America; 2 Walter Reed Army Institute of Research Europe - Middle East (WRAIR E-ME), Tbilisi, Georgia; University of Kentucky College of Medicine, UNITED STATES OF AMERICA

## Abstract

In order to investigate previously reported expansion of tick-borne pathogenic viruses in Eastern Europe, we conducted this study using pooled ticks collected from various locations in Poland, utilizing Sequence Independent Single Primer Amplification (SISPA) and metagenomic sequencing. We processed 575 *Dermacentor reticulatus* and *Ixodes ricinus* ticks and generated 280 virus assemblies in 20 pools. Viruses representing 28 species or strains classified in 12 families or higher taxonomic ranks were observed. We identified four tick-borne human pathogens including Alongshan virus (ALSV), Tacheng tick virus 1 (TcTV-1), Tacheng tick virus 2 (TcTV-2) and Nuomin virus (NUMV), in 55% of the pools, comprising 19.2% of the assemblies. We detected ALSV in *I. ricinus* ticks, with virus genome segments in complete or near-complete forms, comprising the initial reporting of ALSV from Poland. Further analyses revealed phylogenomic clustering with ALSV strains from Europe and lack of recombination signals among virus genomes. TcTV-1 was detected in 35% of the pools comprising *D. reticulatus* and *I. ricinus* ticks, implicating *I. ricinus in* TcTV-1 transmission for the first time. Maximum likelihood analyses on TcTV-1 and TcTV-2 genome segments indicated separate clustering patterns suggesting geographically-segregated clades. Evidence for NUMV or a closely-related chuvirus in *I. ricinus* ticks was further noted. In conclusion, we identified persistence of previously-documented tick-borne pathogens in Poland as well as additional viruses such as ALSV. Assessment of temporal and spatial patterns for virus circulation and diagnostic assays for these agents is needed. The distribution and public health impact of these pathogens throughout Europe require further investigation.

## Introduction

Viruses transmitted via tick vectors are considered as an escalating threat for human and animal health in Europe [1]. An expansion of the affected regions and incidence of the main tick-borne viral agents of public concern; namely Crimean-Congo hemorrhagic fever virus (CCHFV) (*Nairoviridae*, *Orthonairovirus haemorrhagiae*) and tick-borne encephalitis virus (TBEV) (*Flaviviridae*, *Orthoflavivirus encephalitidis*) are well-documented [2,3]. In addition to these viruses with potentially severe outcomes, there are many other circulating tick-borne viruses, detected in both ticks and wild mammalian species, and several have the potential to impact the health of humans, domestic animals and wildlife, such as African swine fever virus (genus *Asfivirus*), louping ill virus (*Orthoflavivirus loupingi*) and other agents such as Greek/Spanish goat encephalitis viruses [1,4]. The capacity of ticks to transmit pathogens across multiple life stages via several blood meals generates complex epidemiological relationships with reservoir and spillover hosts [1]. Moreover, the impact of climate change, land use and human activities seem to have affected tick population dynamics, facilitating transmission and virus emergence [5]. A spillover event by a novel virus or expansion of known agents may be overlooked until the occurrence of an epidemic or clustering of symptomatic infections involving humans or domestic animals. Therefore, surveillance is of utmost importance for timely identification of newly-introduced pathogens and subsequent assessment of potential public health threat posed by these agents.

The spectrum of viruses harbored by ticks has been best explored by the advancing metagenomic sequencing (MS) techniques, where entire populations of microorganisms of the ticks and their blood meals could be analyzed without prior target information [6]. Using these approaches, an extensive range of viruses encompassing several virus families have been described in various tick populations from the Balkans to Norway [7–9]. While it remains unclear whether some of these viruses are insect-specific or transmissible between hosts, they highlight viral diversity and potential interactions of pathogens with the community of microorganisms within the tick microbiome [10]. Supported by MS, recent reports documented the expansion of particular tick-borne pathogenic viruses that have caused outbreaks and human infections in Asia, towards regions in Eastern Europe and the Black Sea region [11,12]. The lack of standardized nucleic acid or serology-based diagnostics significantly impedes the identification of new infections or exposure in areas of probable activity and widespread screening.

A notable shortcoming of the non-targeted MS is the decreased detection sensitivity in targets of low abundance, such as viruses, especially in samples with a vast background of host and environmental nucleic acids, as observed in ticks. This limitation can be circumvented by increasing sequencing depth (and per sample cost in parallel), or enriching for targeting priority pathogens by various approaches [13]. Based on ligation of an asymmetric primer and subsequent amplification, the Sequence Independent Single Primer Amplification (SISPA) method enables enrichment of minute amounts of viral nucleic acids, to be subsequently analyzed [14]. Coupled with MS, the SISPA technique is capable of recovering a wide range of virus sequences and whole genomes from a variety of samples and from those with mixed infections [15,16]. The efficacy of this approach has also been assessed for tick-borne viruses, including CCHFV and Jingmen tick virus [16,17]. In this study, we aimed to perform a follow-up screening utilizing a SISPA+MS approach, in ticks collected at locations with previous detection of tick-borne virus expansion.

## Materials and methods

### Ethics statement

The study does not involve human or animal samples; therefore, no approval from any institutional ethics committee was sought.

### Tick collection

Questing adult ticks were processed for the study previously collected via flagging at four *ad hoc* sites in Poland in 2023. Collected ticks were killed in −20°C freezer prior to shipping. Tick specimens were transported in DNA/RNA Shield at environmental temperature to Walter Reed Army Institute of Research Europe-Middle East (WRAIR E-ME). Upon arrival to WRAIR E-ME the ticks were stored at −20°C. The tick specimens were identified using morphology keys [18], then separated by species, sex, and collection site in preparation for molecular processing. Subsequently, pools of 1–5 ticks were frozen on dry ice and homogenized with 5 mm stainless steel bead (TissueLyser LT, Qiagen). TRIzol reagent (ThermoFisher Scientific) was used to deactivate pathogens and separate DNA and RNA phases. (TRIzol to chloroform 3:1 ratio, centrifugation 12000 x g for 15 min). Separated DNA and RNA phases were mixed with 70% ethanol and commercially available QIAGEN silica spin column kits were used for nucleic acid purification (RNeasy Kit Cat# 74106 and DNeasy blood and tissue Cat# 69506). RNA concertation was measured using Qubit 4 fluorometer (ThermoFisher Scientific). Relatively similar amount of RNA templates from six different regular pools were mixed together in one bigger pool (30 tick maximum) and total amount of 200–500 ng RNA mix were used for SISPA.

### SISPA and MS

Fifteen uL of the total nucleic acids was treated with DNase TURBO (Thermofisher, CA, USA) by incubating it at 37°C for 30 minutes to deplete DNA. The RNA was cleaned using RNAClean XP (Beckman Coulter, USA) and used to synthesize the first complementary DNA (cDNA) strand using SISPA random primers as described previously [14]

(FR26.RV5´-GCCGGAGCTCTGCAGATATCNNNNNN-3´) with Superscript IV first strand synthesis kit (Invitrogen, CA, USA). The second strand cDNA was synthesized by a Klenow reaction (New England Biolabs, MA, USA). Random fragment amplification was done using Phusion High-Fidelity DNA Polymerase master mix (New England Biolabs, MA, USA) with 5 µM of primer FR20.RV (5´-GCCGGAGCTCTGCAGATATC-3´) with the following thermal regimen: one cycle of 98°C for 30 sec, followed by 40 cycles each of 98°C for 10 sec, 50°C for 10 sec and 72°C for 45 sec. A final extension step of 72°C for 10 min was included, followed by a 12°C holding step. Followed by 2nd round random fragment amplification with 100 µM of primer FR20.RV (5´-GCCGGAGCTCTGCAGATATC-3´) with the following thermal regimen: 1 cycle of 98°C for 30 sec, 1 cycle of 50°C for 10 sec and 72°C for 10 min, followed by a 12°C holding step. The random amplicons were cleaned with Agencourt Ampure XP beads (Beckman, CA, USA). The generated DNA libraries were quantified using a Qubit dsDNA HS Assay kit (Invitrogen, CA, USA) and the library size measured on the 4200 TapeStation System (Agilent Technologies, CA, USA). The pooled library was adjusted to 16 pM concentration and spiked with 5% PhiX control (Illumina, CA, USA) and the library was sequenced using the MiSeq Reagent Kit V3 600 cycles on the MiSeq (Illumina, CA, USA).

### Data processing and assembly

Demultiplexed raw Illumina data were adaptor trimmed, and quality filtered using fastp v0.23.3 (--qualified_quality_phred = 15; --unqualified_percent_limit = 40; [19,20]. The cleaned data was then separately de-novo assembled using five different assembly tools: MEGAHIT v1.2.9 [21], Trinity v2.15.1 [22], meta-Spades, metaviral-Spades and rnaviral-Spades (options --meta, --metaviral and --rnaviral, respectively, in Spades v4.0; [23]. Assembled contig headers were then prefixed with their respective assembler using SeqKit [24] and all five assembler outputs were combined and clustered using the cd-hit-est algorithm in CD-HIT [25]; to reduce sequence redundancy and select for the longest contig within clusters of 95% identity. Subsequently, these contigs were classified into viruses using neural network models in DeepMicroClass [26] and geNomad [27]. The quality of classified virus contigs was then assessed using CheckV [28]. Contigs classified as complete or high quality (>90% completeness) were then aligned to the NCBI protein non-redundant (nr) database using Diamond [29] and taxonomically binned using Megan v6.24.20 [30,31]. The analysis workflow is available at https://github.com/rocherbpb/viral_discovery.

### Sequence handling and phylogenetic analysis

Asssemblies were handled using Geneious Prime (v2023.2.1) (Biomatters Ltd., Auckland, New Zealand). Minimap2 and CLUSTALW was used for contig mapping, alignment and pairwise comparisons [32,33]. BLASTn and BLASTp algorithms were used for similarity searches in the NCBI database [34]. Phylogenetic relationships between virus contigs and near relatives were explored using maximum likelihood analysis performed in MEGA v11.0.13 [35]. The optimal model for the phylogenetic and molecular evolutionary analyses in MEGA was determined using the built-in “Find Best DNA/protein-substitution model” Potential genetic exchange and recombination were assessed using the RDP4 (v4.101) [36]. Protein domain and motif searches were performed using the NCBI conserved domain search tool and MOTIF Search in the PFAM database [37,38].

## Results

A total of 575 ticks, identified as *Dermacentor reticulatus* (265, 46.1%) and *Ixodes ricinus* (310, 53.9%), were processed in 20 pools. Two hundred and eighty assemblies were generated, originating from *D. reticulatus* (111, 39.6%) and *I. ricinus* (169, 60.4%) samples (Table 1). The assembled contigs represented viruses from 12 families (237, 84.6%), and those from higher taxonomic ranks (three orders and one realm; 15.4%). Virus contigs classified in *Nairoviridae* (86, 30.7%), *Flaviviridae* (68, 24.3%) and *Phenuiviridae* (58, 20.7%) were the most abundant, followed by those from *Bunyavirales* (28, 10%) and others. A distribution trend according to tick species was observed: *Flaviviridae* and *Phenuiviridae* were predominantly detected in *Dermacentor reticulatus*, whereas *Nairoviridae* and *Bunyavirales* were mainly identified in *I. ricinus* (Table 1). Information on individual pools and virus detection are provided in S1 and S2 Tables.

**Table 1:** Taxonomy and distribution of virus contig counts according to tick species.

Virus family	Virus species	Tick species	
		*D. reticulatus*	*I. ricinus*
*Alphatetraviridae*	Tick alphatetravirus 1 (n = 2)	0	2
*Botourmiaviridae*	*Erysiphe necator* associated ourmia-like virus 16 (n = 1)	0	1
*Chuviridae*	Noumin virus (n = 1)	0	1
*Flaviviridae*	Alongshan virus (n = 4)	0	4
	*Dermacentor reticulatus* pestivirus-like virus 1 (n = 64)	50	14
*Iflaviridae*	*Ixodes scapularis* iflavirus (n = 2)	0	2
*Nairoviridae*	Norwavirus sp. (n = 38)	0	38
	Sulina virus (n = 16)	0	16
	Tacheng tick virus 1 (n = 32)	15	17
*Narnaviridae*	*Plasmopara viticola* lesion associated narnavirus 2 (n = 1)	0	1
	Serbia narna-like virus 3 (n = 6)	0	6
*Partitiviridae*	Jilin partiti-like virus 1 (n = 1)	0	1
*Peribunyaviridae*	*Ixodes scapularis* bunyavirus (n = 1)	0	1
*Phenuiviridae*	Blacklegged tick virus 3 (n = 2)	0	2
	Changping tick virus 1 (n = 2)	0	2
	*Dermacentor reticulatus* uukuvirus (n = 30)	26	4
	Fairhair virus (n = 7)	0	7
	Tacheng tick virus 2 (n = 17)	13	4
*Rhabdoviridae*	Chimay rhabdovirus (n = 7)	0	7
	*Dermacentor reticulatus* rhabdovirus 1 (n = 1)	1	0
	*Rhabdoviridae* sp. (n = 1)	0	1
*Totiviridae*	Tonghua Totiv tick virus 1 (n = 1)	0	1
*Bunyavirales*	Bronnoya virus (n = 28)	0	28
*Mononegavirales*	Norway mononegavirus 1 (n = 2)	0	2
*Tolivirales*	Norway luteo-like virus 2 (n = 1)	0	1
	Norway luteo-like virus 3 (n = 3)	0	3
*Riboviria*	Bole tick virus 4 (n = 8)	6	2
	*Ixodes scapularis* associated virus 3 (n = 1)	0	1
		**111**	**169**

Overall, we assembled contigs of 28 distinct virus species or strains, where *Phenuiviridae* exhibited the most diverse virus content, with five distinct viruses. Other than *Phenuiviridae*, each taxonomic tier comprised 1–3 viruses (Table 1). All viruses were isolated or detected in ticks, except for *Plasmopara viticola* lesion associated narnavirus, associated with the virome of grapevine downy mildew lesions [39]. Among tick-associated viruses, four viruses documented as tick-borne human pathogens were observed, comprising Alongshan virus (ALSV; *Flaviviridae*), Tacheng tick virus 1 (TcTV-1; *Nairoviridae*, *Orthonairovirus tachengense*), Tacheng tick virus 2 (TcTV-2; *Phenuiviridae*, *Uukuvirus tachengense*) and Nuomin virus (NUMV; *Chuviridae*) [40–43]. They were detected in 11 tick pools in total (11/20, 55%): 7 (77.8%) comprising *D. reticulatus* and 4 (36.4%) of the *I. ricinus* pools (Table 2). Tick-borne pathogenic virus contigs comprised 19.2% (54/280) of the generated assemblies. All tick samples with detectable pathogens originated from the identical collection site (S2 Table).

**Table 2:** Overview of the tick-borne pathogenic virus contigs.

Species	Sample ID (#)	ALSV ^a^	TcTV-1 ^b^	TcTV-2 ^c^	NUMV ^d^	*Total* (%)
*D. reticulatus*	VCT35 (16)	–	3	2	–	5 (31.2)
	VCT36 (9)	–	–	3	–	3 (33.3)
	VCT37 (13)	–	–	3	–	3 (23.1)
	VCT38 (10)	–	4	–	–	4 (40)
	VCT39 (19)	–	6	3	–	9 (47.3)
	VCT40 (12)	–	2	1	–	3 (25)
	VCT41 (9)	–	–	1	–	1 (11.1)
*I. ricinus*	VCT43 (16)	–	6	3	–	9 (56.2)
	VCT44 (8)	–	1	–	–	1 (12.5)
	VCT45 (19)	–	10	1	–	11 (57.8)
	VCT50 (41)	4	–	–	1	5 (12.1)
	*Total* (280)	4 (1.4)	32 (11.4)	17 (6.1)	1 (0.3)	54 (19.2)

^a^Alongshan virus;

^b^Tacheng tick virus 1;

^c^Tacheng tick virus 2;

^d^Nuomin virus

### Alongshan virus (ALSV)

**Fig 1:**
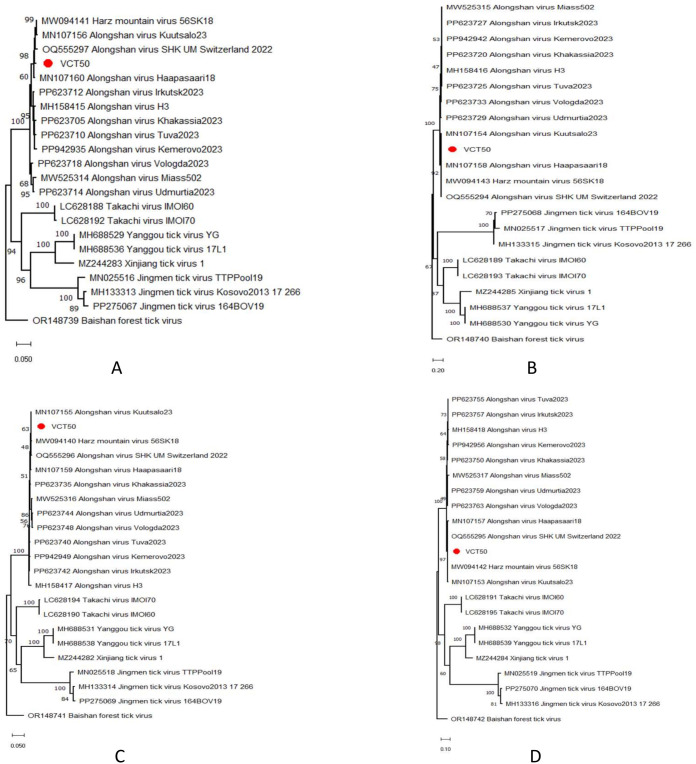
The maximum likelihood trees of the Alongshan virus proteins. The trees are based on NS5-like protein (A: segment 1, 849 amino acids), VP1a/b (B: segment 2, 460 amino acids), NS3-like protein (C: segment 3, 483 amino acids) and VP2/3 (D: segment 4, 757 amino acids) alignments. The trees are constructed using the LG model (segments 1, 3 and 4) or the Jones-Taylor-Thornton model (segment 2), with a discrete Gamma distribution (G) for 500 replications. Sequences obtained in the study are labeled with sample identifiers. Virus strains are indicated by GenBank accession number, name and isolate identifier. Baishan forest tick virus was included as an outgroup.

Four contigs with the preliminary identification of Jingmen group viruses were present in an *I. ricinus* pool. They covered all individual virus genome segments with complete/near-complete coding regions described for the group [44]. Maximum likelihood analyses of putative viral proteins revealed clustering of these sequences with ALSV strains from Germany, Switzerland and Finland within the Jingmen group (Fig 1). Alignment and pairwise comparisons within this subclade showed nucleotide and putative amino acid diversities up to 5.9%, and 1.5%, respectively (S3 Table). We further analyzed potential intra- and interclade recombinations using available ALSV genomes, including those identified in this study. These analyses did not reveal any *in silico* evidence for recombination within the European clade or among geographically-segregated ALSV clades. Conserved domain searches on individual genome segments of ALSV-Poland revealed the N-terminal helicase domain (cl28899) on putative NS3 protein (amino acids: 292–444); as well as viral Cap-specific methyltransferase (cl41719; amino acids: 21–244) and conserved RNA-dependent RNA polymerase catalytic core domain (cl40470; amino acids: 345–778) on putative NS5 protein.

### Tacheng tick virus 1 (TcTV-1) and nairoviruses

**Fig 2:**
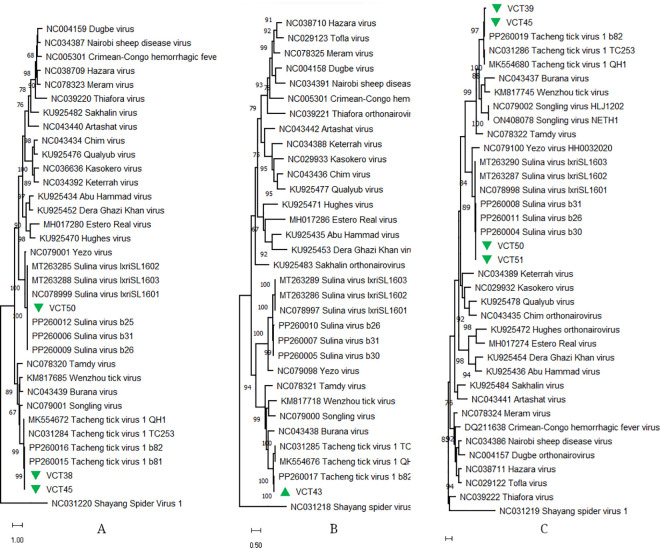
The maximum likelihood trees of the *Orthonairovirus* proteins. The trees are based on replicase (A: L segment, 500 amino acids), glycoprotein precursor (B: M segment, 213 amino acids) and nucleoprotein (C: S segment, 180 amino acids) alignments, and constructed using the Jones-Taylor-Thornton model for 500 replications. Sequences obtained in the study are labeled with sample identifiers. Virus strains are indicated by GenBank accession number, name and isolate identifier. Shayang spider virus 1 was included as an outgroup.

We detected TcTV-1 in four *D. reticulatus* and three *I. ricinus* pools, comprising 35% of the pools and representing 11.4% of the generated contigs (Tables 1 and 2). Within a size range of 502−1940 nucleotides, all three virus genome segments (L, M and S) were identified in two samples, whereas contigs of two segments (L and M/S) were detectable in four (S2 Table). In maximum likelihood trees, all sequences clustered with TcTV-1 strains among nairoviruses (Fig 2). In addition to TcTV-1, sequences of a related nairovirus, Sulina virus (SULV, *Orthonairovirus sulinaense*), were identified in four *I. ricinus* pools. Similar to TcTV-1, three genome segments were detected, with complete coding regions recovered for L and S segments. Sharing a common ancestor with Yezo virus, another tick-borne human pathogenic nairovirus [45], these sequences remained within the SULV cluster in the maximum likelihood analyses, to further group with viruses from Latvia (Fig 2).

Finally, *Norwavirus* sp. (family *Nairoviridae*, genus *Norwavirus*) sequences were detected in eight *I. ricinus* pools (40%), comprising 38 contigs (13.6%) encompassing nucleocapsid and polymerase coding regions on S and L segments, respectively. In infected pools, sequences with identities to various strains within the genus were noted, including Grotenhout virus, Pustyn virus, Norway nairovirus 1 and *I. ricinus* orthonairovirus (S2 Table). Maximum likelihood trees build on complete coding regions recovered in an *I. ricinus* pool placed these viruses in the same clade, without phylogenetic support for individual virus description, distinct from the documented human pathogen, Beiji nairovirus (BJNV) (S1 Fig) [46].

### Tacheng tick virus 2 (TcTV-2) and phenuiviruses

**Fig 3:**
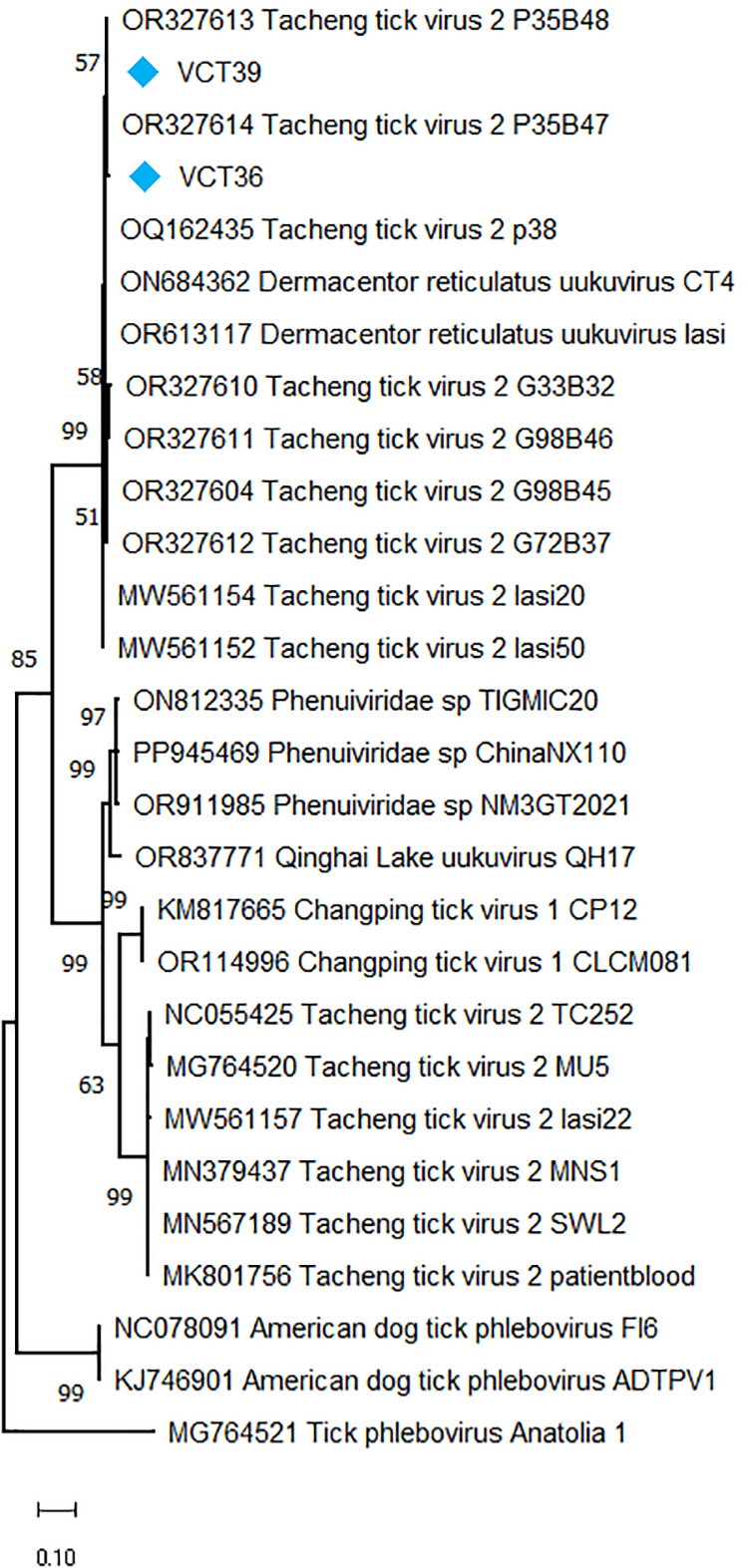
The maximum likelihood tree of the Phenuivirus replicase. The tree is based on an alignment of 324 amino acids, and constructed using the LG model with a discrete Gamma distribution (G) and invariable sites **(I)**, for 500 replications. Sequences obtained in the study are labeled with sample identifiers. Virus strains are indicated by GenBank accession number, name and isolate identifier. Tick phlebovirus Anatolia 1 was included as an outgroup.

Six *D. reticulatus* and two *I. ricinus* pools produced TcTV-2 assemblies, comprising 40% of the pools and 6.1% of the assemblies. The sequences originated from the L segment of the virus genome with a size range of 242–2034 nucleotides. In the maximum likelihood analyses of the putative viral replicase sequences, they were grouped with TcTV-2 sequences previously reported from Poland and Georgia, forming a separate virus clade distinct from those documented in Asia (Fig 3). Along with particular TcTV-2 strains, this clade further included *Dermacentor reticulatus* uukuvirus (DRUV), another closely-related phenuivirus, originally reported from the Balkan Peninsula, with unexplored medical or veterinary impact [8]. DRUV was also present in our cohort, in seven *D. reticulatus* and two *I. ricinus* pools, some of which with detectable TcTV-2 (S2 Table). We generated separate alignments and built maximum likelihood tree due to the lack of overlaps with TcTV-2 sequences, which revealed these sequences to belong in the same clade (S2 Fig).

We further identified other tick-associated phenuiviruses in the samples, including those classified under the genera *Phlebovirus* (Changping tick virus 1) or *Ixovirus* (Blacklegged tick virus 3, and Fairhair virus) [47]. Maximum likelihood trees generated on L and S segment sequences obtained from an *I. ricinus* pool showed grouping with closely-related viruses (S3 Fig).

### Nuomin virus (NUMV) and bronnoya virus

As the sole representative of the family *Chuviridae*, a single contig of NUMV was detected in an *I. ricinus* pool, with nucleotide and putative amino acid identities of 79.7% and 96.8%, respectively. In the maximum likelihood analysis, it remained within the NUMV/ Lesnoe mivirus clade as a distinct taxon (S4 Fig).

Bronnoya virus (BroBV), a currently unclassified *Bunyavirales* genus described in *Ixodes* sp. ticks from Europe [9], was also detected in five *I. ricinus* pools and comprised 10% (28) of the assemblies (Table 1). We identified two previously-described genomic segments of the virus genome, namely L and M, encoding for the putative replicase and glycoprotein, respectively (S2 Table). Maximum likelihood trees showed the grouping of the L segment sequence within the BroBV clade, while the M segment grouped distinctly with a particular strain Croatia (S5 Fig) [8].

## Discussion

Aiming to investigate previously reported expansion of tick-borne pathogenic viruses in Eastern Europe [11], we conducted this study using pooled ticks collected from various locations in Poland, employing a SISPA+MS strategy for virus detection and genome characterization. We processed 575 *D. reticulatus* and *I. ricinus* ticks and generated 280 assemblies in 20 pools. Virus contigs representing 28 species or strains classified in 12 families or higher taxonomic ranks were assembled, where members of the families *Nairoviridae*, *Flaviviridae* and *Phenuiviridae* comprised 75.7% of the total contigs. We identified four tick-borne human pathogens, ALSV, TcTV-1, TcTV-2 and NUMV, in 55% of the screened tick pools and 19.2% of the assemblies (Table 2).

We detected ALSV in a pool of *I. ricinus* ticks, with all four virus genome segments in complete or near-complete forms, comprising the initial reporting of ALSV from Poland. Classified in the Jingmenvirus group of the family *Flaviviridae*, ALSV is associated with a non-specific tick-borne disease in humans, sometimes called as Alongshan fever, presenting as a febrile disease with headache, skin rash, myalgia, arthralgia, depression, and coma [44]. It has been speculated that ALSV employs similar mechanisms documented in tick-borne flavivirus related central nervous system disease, with particular virus non-structural proteins altering innate immune responses [44,48]. Several *Ixodes*, *Dermacentor* and *Haemaphysalis* ticks including *I. ricinus* have been documented to harbor ALSV, with evidence of virus exposure in sheep, cattle, and deer [46,49–52]. Although clinical cases have solely been described from China, ALSV was reported in ticks from many Eurasian countries including Russia, Finland, France, Germany, Serbia and Switzerland. Phylogeographic analyses identified three major clades consistent for each genome segment where clade 1 includes genomes in or near Europe, clade 2 around the Russia-Kazakhstan border and clade 3 from eastern part of Asia [44]. Our findings indicate ALSV from Poland to belong in the European clade (clade 1). A comparable pattern of diversification has been observed in other endemic tick-borne flaviviruses such as JMTV and tick-borne encephalitis virus (TBEV), with established European, Siberian, and East Asian clades and different clinical outcomes [53]. It remains to be elucidated whether ALSV strains from different geographical regions vary in pathogenicity and lack of diagnosed cases from Europe could be attributed to virus clades. We could not demonstrate any evidence of recombination among ALSV clades including the newly-described genomes from Poland, suggesting limited genetic exchange among clades or strains, further supported by the lack of eminent viral diversity or mutational hotspots in previous analyses [44]. Interestingly, in a recent *in silico* virus investigation on publicly available tick transcriptome data, ALSV was reported in field-collected ticks from Spain [54], suggesting a more widespread virus distribution in the European continent.

Another tick-borne human pathogen identified in the study is TcTV-1, detected in 35% of the pools comprising *D. reticulatus* and *I. ricinus* ticks. We observed sequences of various sizes representing all virus genome segments in positive pools. TcTV-1 is among the emerging tick-borne nairoviruses, originally described using MS [55]. Human cases of TcTV-1 mainly present with tick bite associated febrile disease and skin rash. In symptomatic cases, the virus was detectable in throat swabs and urine, suggesting possible transmission by direct contact with body fluids, as observed in CCHFV. Co-infection of TcTV-1 and *Rickettsia* was documented in a case with febrile disease and meningitis, suggesting probable CNS involvement during infection [56]. Particular wild and domestic animals seem to be susceptible to TcTV-1 infections, as exposure is documented in sheep, cattle and human populations with detectable viral genomes in great gerbils (*Rhombomys opimus*) [57]. The main vector of TcTV-1 was considered as *Dermacentor* spp., ticks, due virus genome being identified in several species within the genus including *D. marginatus*, *D. silvarum*, *D. nuttalli* and *D. reticulatus* [11,12,41]. However, the virus was further detectable in *Hyalomma asiaticum* and *Hyalomma aegyptium* ticks in various regions, suggesting a wider range of ticks as potential hosts [41,58]. Here, we report TcTV-1 in *I. ricinus* ticks, documenting this important vector species to host yet another tick-borne pathogen [1]. We have previously reported partial and whole genome sequences of the TcTV-1 in *D. reticulatus* ticks from Poland, collected during 2021 at overlapping sites in this study [11,12]. These findings indicate ongoing viral activity in the region, involving *Ixodes* as well as *Dermacentor* ticks. Our analyses demonstrated sequences of individual genome segments of TcTV-1 to form a separate cluster with the prototype virus genome from Poland, representing a geographically segregated clade. Overall, the TcTV1-Poland complete genome topology was observed as similar to those reported from China and should possess similar or comparable pathogenicity as viruses isolated from symptomatic individuals [12]. These findings warrant a wider screening for TcTV-1 in Europe to detect possible infections in humans.

We detected other nairoviruses in the samples, including SULV, which was described in *I. ricinus* ticks from Romania [59], and further detected in Latvia [12], with and closely-related viruses reported in France [60]. Moreover, sequences of *Norwavirus* sp. were identified in *I. ricinus*, comprising 13.6% of the assembled contigs. According to the current taxonomy, the *Norwavirus* genus of the *Nairoviridae* family includes the Grotenhout virus as *Grotenhout norwavirus* species, and the other closely-related viruses remain unclassified [61]. Norwavirus genomes consists of two segments that encode for the nucleoprotein (S segment) and replicase (RNA-dependent RNA polymerase, L segment), a unique feature among nairoviruses. So far, only BJNV (*Norwavirus beijiense*) in the genus have been directly associated with tick-borne human disease [46]. The virus sequences generated in this study shared identities with several unclassified members of the genus, including Grotenhout and Pustyn viruses, Norway nairovirus 1 and *I. ricinus* orthonairovirus, previously reported in *I. ricinus* from Belgium, Bulgaria, Norway, France, Romania as well as Poland [7,11,62], without phylogenomic support for any distinct species. Therefore, multiple, closely-related viruses could be in circulation in our sampling locations, which is comparable to the previous screening findings [11]. Evidently, norwaviruses are widespread among European *I. ricinus* populations.

We generated sequences of *Phenuiviridae* in the samples, comprising one of the abundant virus families observed in the study. Among these, we detected the tick-borne pathogen TcTV-2 in pools of both tick species examined (Table 2). Following initial description in field-collected ticks, human infections of TcTV-2 were documented in China, associated with clinical symptoms of undifferentiated febrile disease and meningitis-like signs [42,63]. As observed for TcTV-1, viral genomes could be detected in blood, urine, and throat swabs of the affected individuals. Red foxes (*Vulpes vulpes*) and Asian badgers (*Meles leucurus*) are suggested as potential virus reservoirs in nature [64]. TcTV-2 was detected in several tick species including *D. marginatus*, *D. niveus*, *D. nuttalli*, *D. silvarum*, and *H. asiaticum* ticks from China [42,65,66]; in *H. scupense* and *H. anatolicum* from Kazakhstan) [67]; in *D. marginatus* and *H. marginatum* from Turkey [68,69] and *D. reticulatus* from Romania [70]. In particular tick species, evidence of transovarial, transstadial or horizontal virus transmission is documented [42,68,69]. Previously, we reported TcTV-2 using targeted screening and MS in *D. marginatus*, *D. reticulatus* and *Haemaphysalis* spp. from Georgia, Poland and Ukraine [11]. Comparable with our previous findings, maximum likelihood analyses in this study showed two distinct TcTV-2 clades, one comprising sequences from China and Turkey, the other from Georgia, Poland, with the sequences from Romania represented in each clade (Fig 3). Interestingly, the second clade further included DRUV, which was detected in this study and grouped in the same clade as well (S2 Fig). Due to lack of complete virus sequences and overlapping regions in alignments, we could not further analyze phylogenomic relations among these viruses. Nevertheless, their zones of circulation seem to overlap in Eastern Europe and the potential pathogenicity of the viruses in this clade needs further investigation. TcTV-2 and DRUV possess L and S genome segments, seemingly lacking the glycoprotein encoding M segment observed in typical phenuiviruses [8,42]. No evidence of recombination could be demonstrated among selected TcTV2/DRUV genomes [11].

In a pool of *I. ricinus* ticks, a single NUMV contig was generated, which was placed within the NUMV/ Lesnoe mivirus clade (unclassified *Chuviridae*) in maximum likelihood analysis. Chuvirus genomes comprise single-stranded negative sense RNA in segmented, unsegmented, linear or circular topologies [55,71]. In addition to ticks, they are found in several insects, barnacles, decapod crustaceans and reptiles globally. So far, NUMV is the only chuvirus with documented health impact [43,72]. It is identified in case of febrile disease with tick bite history, sometimes with elevated liver transaminases. It is in several common tick species, including *I. persulcatus*, *D. nuttalli*, *Haemaphysalis concinna* and *Hae. longicornis* [43]. Further surveillance is needed to understand the presence and distribution of chuviruses and NUMV in Europe.

Our screening further revealed several tick-associated viruses, where related strains had been previously described in tick virome investigations throughout Europe including BroBV, detected in 20% of the pools and in *I. ricinus* ticks. BroBV and closely-related viruses are recently described viruses associated with *Ixodes* sp. ticks, with a bi-segmented RNA genome lacking the small segment that encodes the viral nucleoprotein present in the majority of Nairo and Phenuiviruses [9]. BroBV genomes are divergent among bunyaviruses and were proposed to represent a separate family in the order *Bunyavirales* [9]. They are found in *I. ricinus* viromes from Croatia, France, Norway, Romania and Russia, and further detected in a *Ixodes scapularis*-derived cell line, without serological evidence of exposure was observed in small ruminants [7–9,73]. Understanding BroBV distribution and impact in *I. ricinus* populations require further research.

Particular shortcomings of this study need to be addressed. The samples were collected in limited number of sites encompassing four provinces, selected to overlap with previous sampling locations with virus detection. For further assessment of tick species involved in virus circulation, we selectively included previously screened tick species predominant in the area. An alternative approach, utilizing SISPA was preferred to detect and characterize viruses. We tested the samples in relatively larger pool sizes, to accommodate a higher number of individuals in the screening. Overall, we could identify not only the previously detected TcTV-1 and TcTV-2, but additional viruses such as ALSV, reported to circulate and cause human infections in Eurasia. These findings demonstrate that the recently-described pathogens; namely TcTV-1, TcTV-2 and ALSV are present in Poland and presumably in the neighboring northeastern European countries. Further studies to assess temporal and spatial patterns for virus circulation and diagnostic assays for these agents are needed. Awareness in medical and public health officials to help to identify potential cases is also of importance. The distribution and public health impact of these pathogens throughout Europe require assessment.

## Supporting information

S1 Fig The maximum likelihood trees of the *Norwavirus* and *Orthonairovirus* proteins. The trees are based replicase (A: L segment, 4765 amino acids), and nucleoprotein (B: S segment, 528 amino acids) alignments, and constructed using the LG model with a discrete Gamma distribution (G) for 500 replications. Sequences generated in the study are labeled with sample identifiers. Virus strains are indicated by GenBank accession number, name and isolate identifier. Tacheng tick virus 1 was included as an outgroup.
(PDF)

S2 Fig The maximum likelihood tree of the *Dermacentor reticulatus* uukuvirus (DRUV) replicase. The tree is based on an alignment of 419 amino acids, and constructed using LG model with a discrete Gamma distribution (G) for 500 replications. Sequences generated in the study are labeled with sample identifiers. Virus strains are indicated by GenBank accession number, name and isolate identifier. Tick phlebovirus Anatolia 1 was included as an outgroup.
(PDF)

S3 Fig The maximum likelihood trees of the Phenuivirus proteins. The trees are based on replicase (A: L segment, 1041 amino acids), and nucleoprotein (B: S segment, 170 amino acids) alignments, and constructed using LG model with a discrete Gamma distribution (G) and invariable sites (I) for 500 replications. Sequences generated in the study are labeled with sample identifiers. Virus strains are indicated by GenBank accession number, name and isolate identifier. Sandfly fever Naples virus was included as an outgroup.
(PDF)

S4 Fig The maximum likelihood tree of the Nuomin virus (NUMV) replicase. The tree is based on an alignment of 268 amino acids, and constructed using LG model with a discrete Gamma distribution (G) and invariable sites (I) for 500 replications. The sequence generated in the study is labeled with the sample identifier. Virus strains are indicated by GenBank accession number, name and isolate identifier. Bole tick virus 3 was included as an outgroup.
(PDF)

S5 Fig The maximum likelihood tree of the Bronnoya virus (BroBV) proteins. The trees are based on replicase (A: L segment, 2513 amino acids), and glycoprotein precursor (B: M segment, 855 amino acids) alignments, and constructed using LG model with a discrete Gamma distribution (G) and invariable sites (I) for 500 replications. Sequences generated in the study are labeled with sample identifiers. Virus strains are indicated by GenBank accession number, name and isolate identifier. Volzhskoe tick virus was included as an outgroup.
(PDF)

S1 Table Classification and distribution of virus contigs in individual tick pools. (XLSX)

S2 Table Information and listing of the virus contigs in pooled tick samples. Collection sites are listed below the table.
(XLSX)

S3 Table Pairwise comparisons of the aligned Alongshan virus sequences. Sequence similarity is expressed as percent identity.
(XLSX)

S1 File SupplementaryTablesRevised_KE. (XLSX)
