# Supplementary figures and images for: Ongoing circulation of emerging tick-borne viruses in Poland, Eastern Europe
Source: PLoS One. 2025 Sep 3;20(9):e0330544. doi: 10.1371/journal.pone.0330544 (PMC12407414; doi:10.1371/journal.pone.0330544)

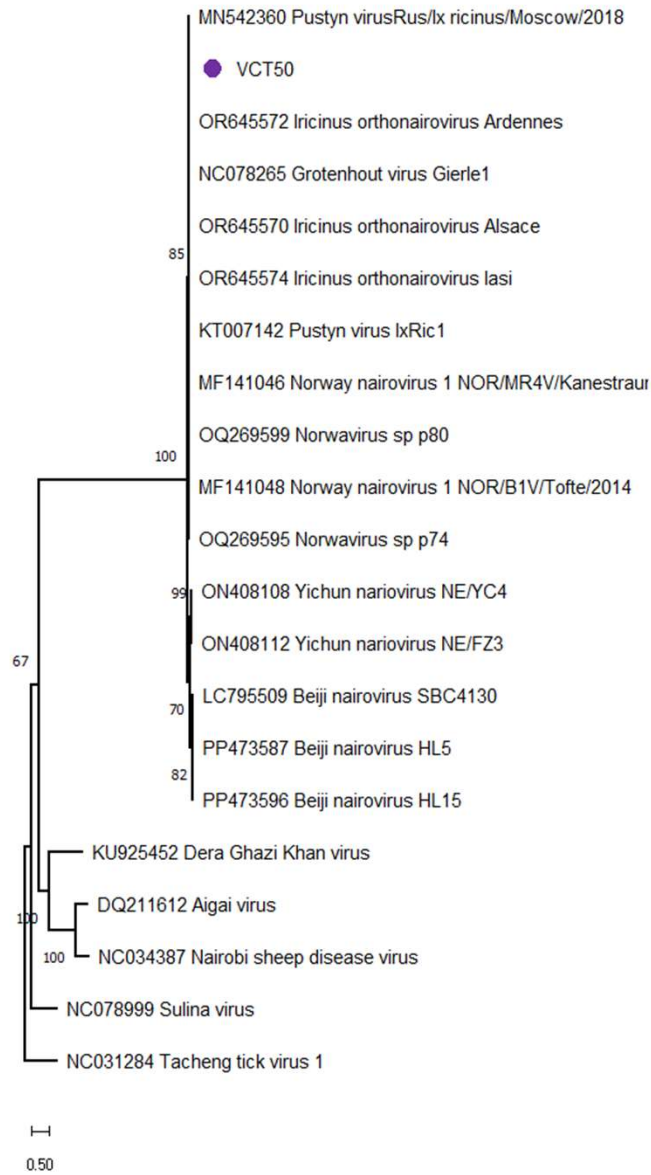

**A**

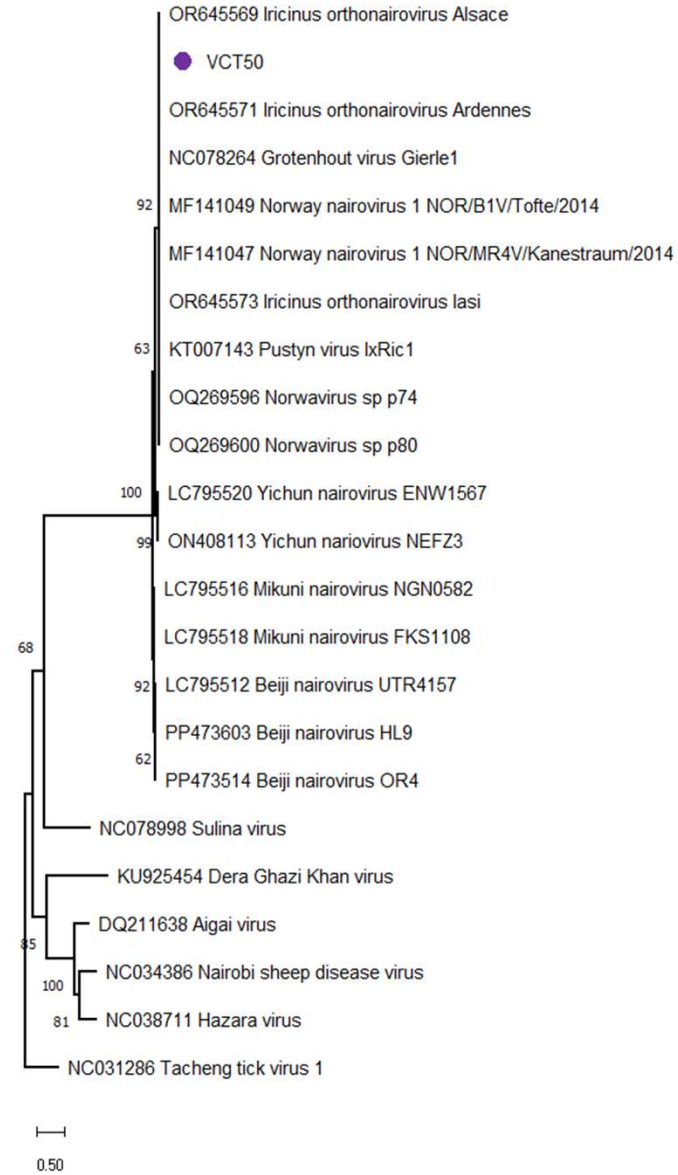

**B**

Supplement: S1 Fig — The trees are based replicase (A: L segment, 4765 amino acids), and nucleoprotein (B: S segment, 528 amino acids) alignments, and constructed using the LG model with a discrete Gamma distribution (G) for 500 replications. Sequences generated in the study are labeled with sample identifiers. Virus strains are indicated by GenBank accession number, name and isolate identifier. Tacheng tick virus 1 was included as an outgroup. (PDF) [file pone.0330544.s001.pdf]

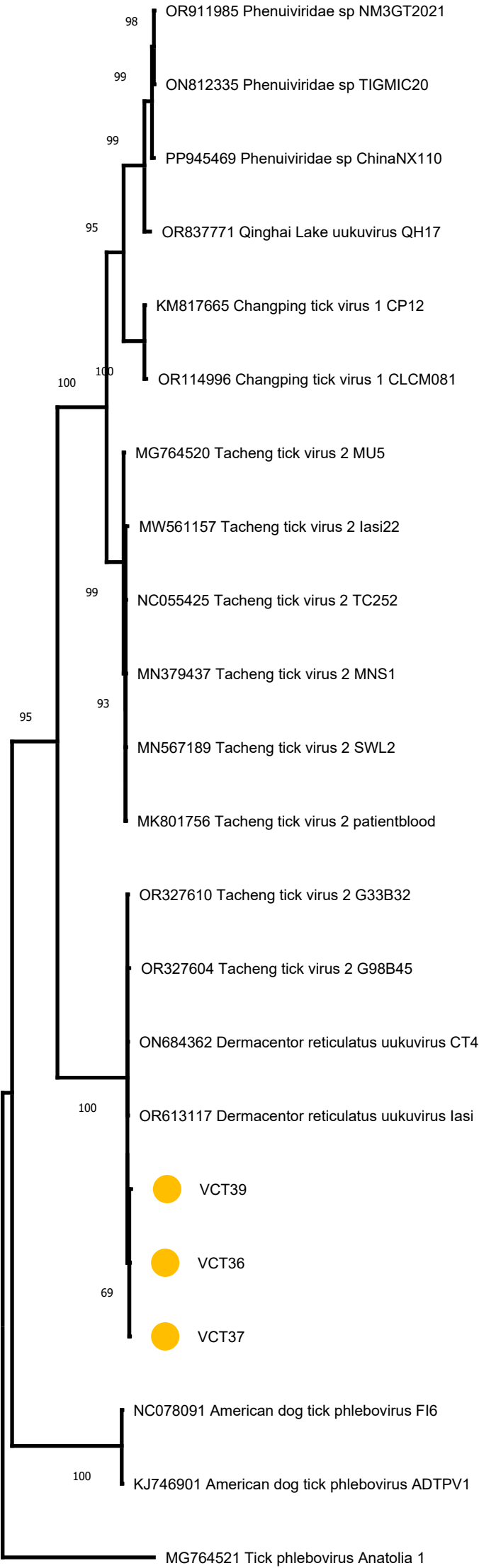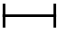

0.20

Supplement: S2 Fig — The tree is based on an alignment of 419 amino acids, and constructed using LG model with a discrete Gamma distribution (G) for 500 replications. Sequences generated in the study are labeled with sample identifiers. Virus strains are indicated by GenBank accession number, name and isolate identifier. Tick phlebovirus Anatolia 1 was included as an outgroup. (PDF) [file pone.0330544.s002.pdf]

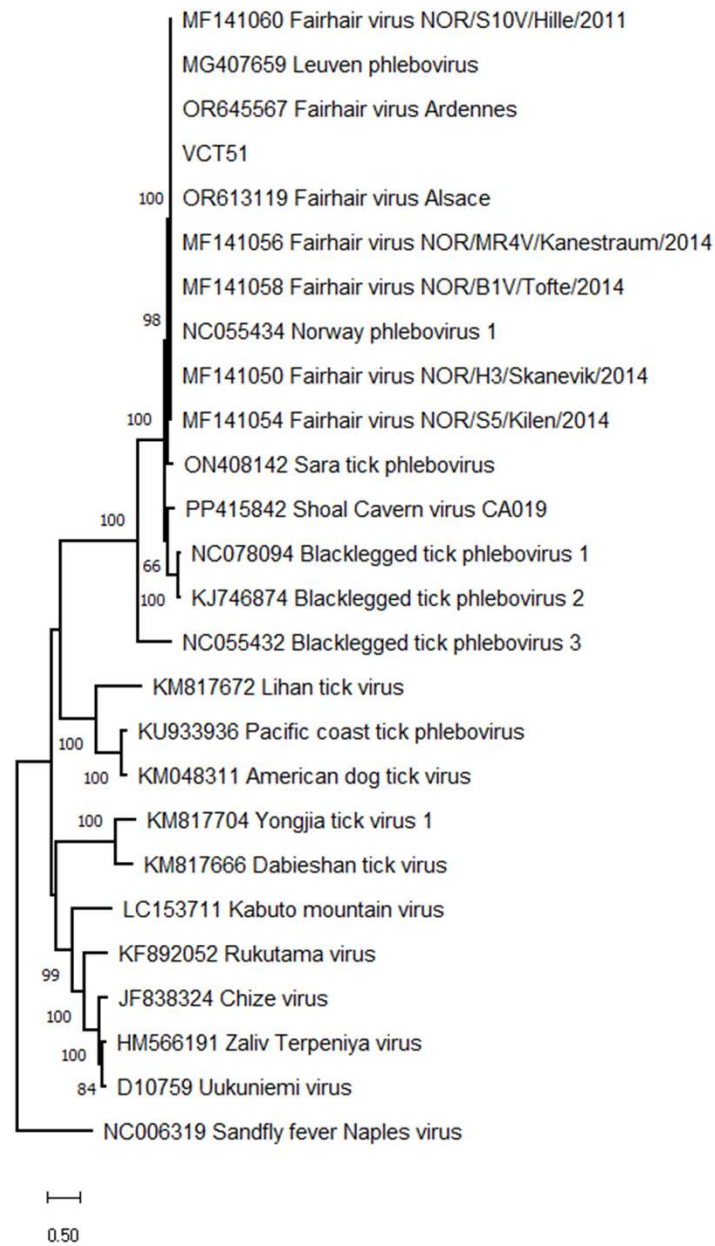

**A**

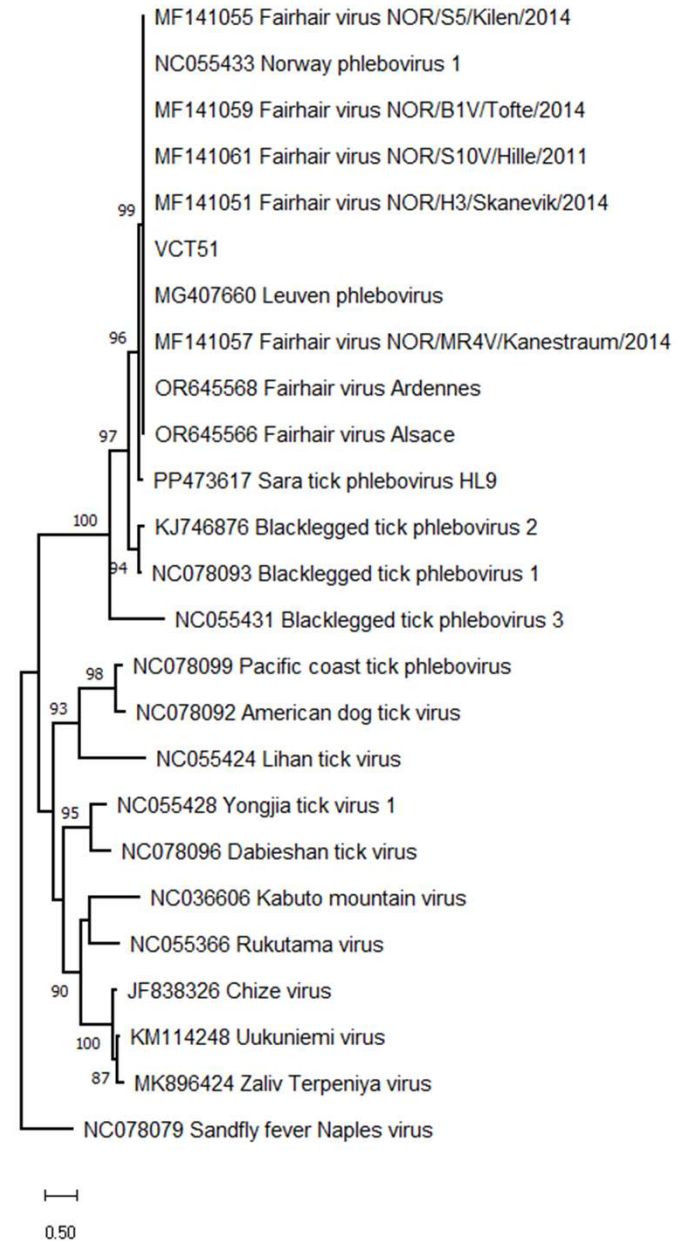

**B**

Supplement: S3 Fig — The trees are based on replicase (A: L segment, 1041 amino acids), and nucleoprotein (B: S segment, 170 amino acids) alignments, and constructed using LG model with a discrete Gamma distribution (G) and invariable sites (I) for 500 replications. Sequences generated in the study are labeled with sample identifiers. Virus strains are indicated by GenBank accession number, name and isolate identifier. Sandfly fever Naples virus was included as an outgroup. (PDF) [file pone.0330544.s003.pdf]

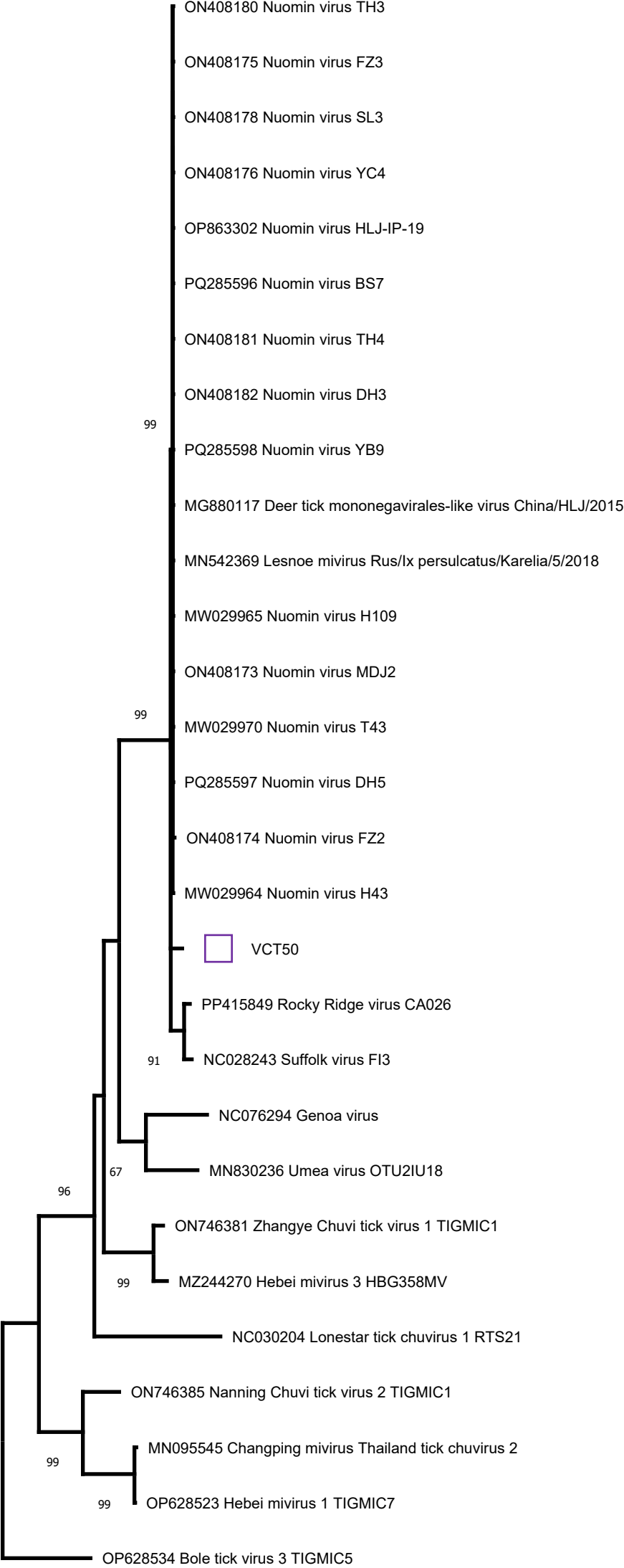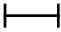

0.10

Supplement: S4 Fig — The tree is based on an alignment of 268 amino acids, and constructed using LG model with a discrete Gamma distribution (G) and invariable sites (I) for 500 replications. The sequence generated in the study is labeled with the sample identifier. Virus strains are indicated by GenBank accession number, name and isolate identifier. Bole tick virus 3 was included as an outgroup. (PDF) [file pone.0330544.s004.pdf]

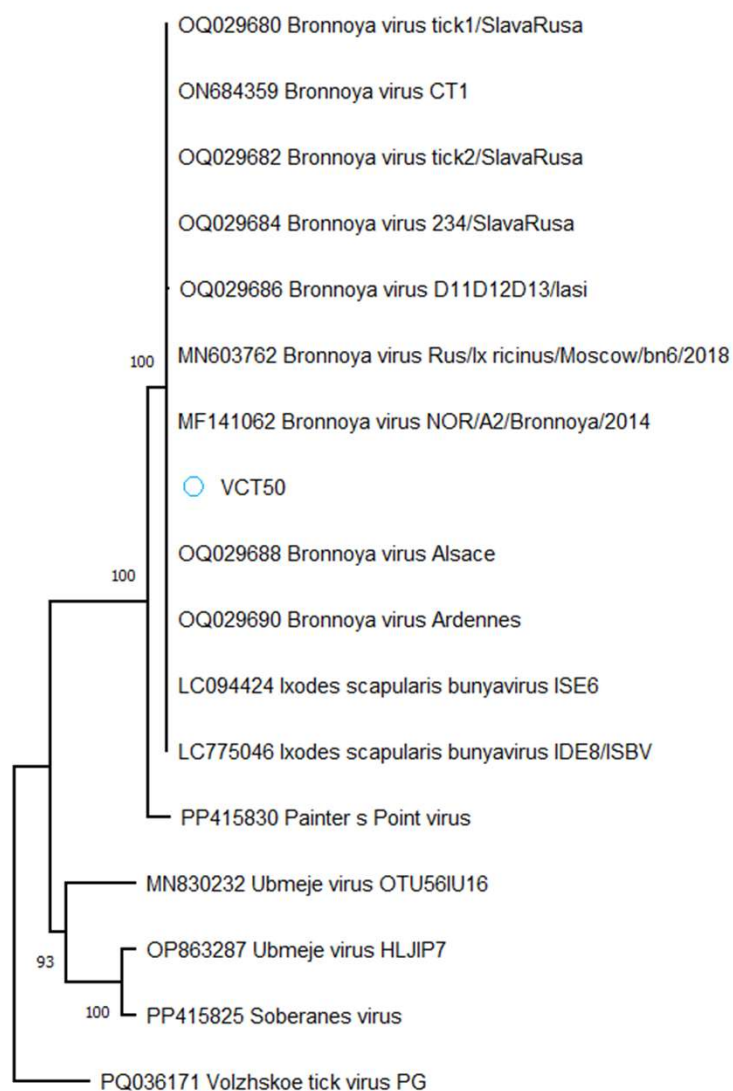

0.20

**A**

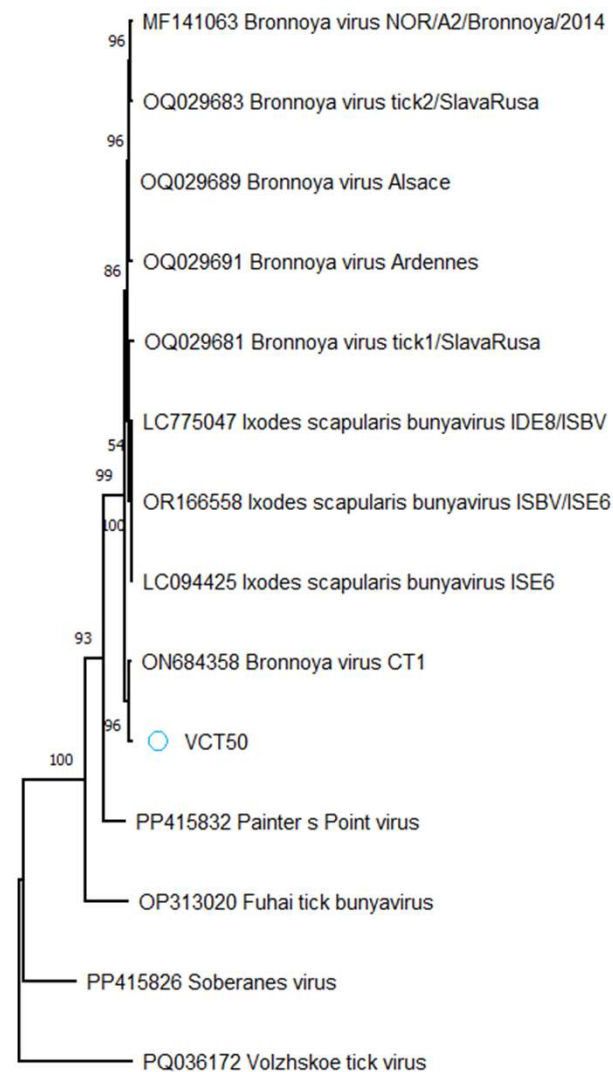

0.20

**B**

Supplement: S5 Fig — The trees are based on replicase (A: L segment, 2513 amino acids), and glycoprotein precursor (B: M segment, 855 amino acids) alignments, and constructed using LG model with a discrete Gamma distribution (G) and invariable sites (I) for 500 replications. Sequences generated in the study are labeled with sample identifiers. Virus strains are indicated by GenBank accession number, name and isolate identifier. Volzhskoe tick virus was included as an outgroup. (PDF) [file pone.0330544.s005.pdf]
